# Welcome to Automated Experimentation: a new open access journal

**DOI:** 10.1186/1759-4499-1-1

**Published:** 2009-10-29

**Authors:** David Robertson, Siu-wai Leung, Dietlind Gerloff

**Affiliations:** 1 School of Informatics, University of Edinburgh, 10 Crichton Street, Edinburgh EH8 9AB, UK; 2 Institute of Chinese Medical Sciences, University of Macau, Av. Padre Tomas Pereira, Macao, PR China; 3 Biomolecular Engineering Department, University of California Santa Cruz, 1198 High Street, Santa Cruz, CA 95064, USA

## Abstract

Modern experimental science provides more opportunities for yet larger series of experiments. Demand for experimental results also has become more diverse, requiring results that have direct connections to systems outside the laboratory. With this has come an ability to automate many areas of experimental science, not only the experiments themselves but also the larger processes that contribute to experimentation and analysis more broadly. As automated experimentation becomes more widely used and understood, we launch this journal to provide a proper publication channel for this new breed of interdisciplinary research as well as a bridge to all significant groundwork research that would facilitate possible automated experimentation. With this in mind, we are interested in publishing all kinds of research into scientific experimentation, including research where the potential for automation is at proof or concept or early deployment stage.

## Editorial

The popular image of traditional science involves researchers toiling laboriously over laboratory experiments, driven by the desire to establish definitive and permanent results. Modern experimental science provides more opportunities for even larger series of experiments. Demand for experimental results has also become more diverse, requiring results that have direct connections to systems outside the laboratory. With this has come an ability to automate many areas of experimental science, not only the experiments themselves but also the larger processes that contribute to experimentation and analysis more broadly. For example:

• Formal process languages allow rapid replication and enactment of experimental designs.

• Service and grid architectures permit experimental data to be shared in large volumes.

• Roboticised laboratory systems permit fast translation of experiments to the physical world.

• Verification methods allow experimental protocols to be more rigorously tested.

• Systems for maintaining experimental context, such as provenance of data.

• Systems to chart broader requirements and argumentation surrounding experiments.

Automation provides many opportunities to conduct traditional experiments more efficiently but, more fundamentally, it allows us to question whether traditional experimentation is the only practical style of experimentation. By traditional experimentation we mean the process of identifying a hypothesis to test; setting up a controlled experiment that tests the hypothesis under specific conditions; then generalising the results of the experiment to confirm/falsify the broader hypothesis. In many situations this traditional experimental method fails because experimental variables are too numerous or complex to control; the experiment is not extensive enough to be convincing; or the generalisation from specific experiments is unjustified.

Automation offers scientists ways of understanding experimental science that avoid these methodological flaws. Rapid synthesis and verification of experimental designs allows more complex experiments to be built and deployed while controlling (or at least better understanding) experimental complexity. By bringing experiments closer to their subjects on a large scale and by taking advantage of the scale of current computational infrastructure more extensive experiments can be performed. Problems with generalisation can be controlled by augmenting results with meta-data on provenance or they can be avoided by rapid re-creation of experiments to cover new situations as they arise.

All of these are comparatively new areas of engineering and most have been developed piecemeal in response to the demands of narrow domains or the opportunities presented by specific technologies. These pioneering efforts are analogous to early experiments in traditional science; they provide examples of what can be done. However, these efforts must then be understood more abstractly in order to become commonplace.

As automated experimentation becomes more widely used and understood, we launch this journal to provide a proper publication channel for this new breed of interdisciplinary research as well as a bridge to all significant groundwork research that would facilitate possible automated experimentation. With this in mind, we are interested in publishing all kinds of research into scientific experimentation, including research where the potential for automation is at proof, concept or early deployment stage. Example areas are listed in the journal's "About" page [1].

Our aim is to bring together the diverse forms of automated experimentation and, by studying them in an abstract but practical way, understand how they generalise across domains. In this way we hope *Automated Experimentation *will promote new styles of computationally inspired experimental thinking.

## Competing interests

The authors declare that they have no competing interests.

## Authors' contributions

DR wrote the manuscript. SL and DG revised and proofread the manuscript. All authors read and approved the final manuscript.
